# Public Perceptions on the Efficiency of National Healthcare Systems Before and After the COVID-19 Pandemic

**DOI:** 10.3390/healthcare13172146

**Published:** 2025-08-28

**Authors:** Athina Economou

**Affiliations:** Department of Economics, University of Thessaly, 38333 Volos, Greece; aeconomou@uth.gr

**Keywords:** healthcare efficiency, decomposition analysis, doctor trust, health spending, behavioural traits, cultural traits

## Abstract

Background/Objectives: This study examines individual perceptions of national healthcare system efficiency before and after the COVID-19 pandemic across 18 countries grouped into three clusters (the Anglo-world, Europe, East Asia). This paper aims to identify the demographic, socioeconomic, health-related, and macroeconomic healthcare drivers of public assessments, and explain changes in attitudes between 2011–2013 and 2021–2023. Methods: Using individual-level data from the International Social Survey Programme (ISSP) for 2011–2013 and 2021–2023, logistic regression models of perceived healthcare inefficiency are estimated. In addition, the Oaxaca–Blinder decomposition model is adopted in order to decompose the assessment gap between the two periods. Models include a range of individual demographic and socioeconomic characteristics and national healthcare controls (healthcare expenditure, potential years of life lost). Results: Health-related factors, especially self-assessed health and trust in doctors, consistently emerge as predictors of more favourable evaluations across regions and periods. Higher national healthcare expenditure is associated with more positive public views and is the single largest contributor to the improved assessments in 2021–2023. Demographic and socioeconomic variables show smaller regionally and temporally heterogeneous effects. Decomposition indicates that both changes in observed characteristics (notably, expenditure and trust) and unobserved behavioural, cultural, or institutional shifts account for the gap in public healthcare assessments between the two time periods. Conclusions: Public assessments of healthcare systems are primarily shaped by individual health status, trust in providers, and national spending rather than differential demographic and socioeconomic traits. Therefore, policymakers should couple targeted investments in the healthcare sector in order to address adequately public healthcare needs, and strengthen doctor–patient relationships in order to sustain public support. Future research should focus on disentangling the cultural and behavioural pathways influencing healthcare attitudes.

## 1. Introduction

The COVID-19 pandemic has placed unprecedented strain on healthcare systems across the globe, triggering public scrutiny of institutional preparedness [1,2]. While the pandemic acted as a stress test for modern healthcare systems, various studies have examined the efficiency of national healthcare systems among European [3] and OECD (Organization for Economic Cooperation and Development) countries [4]. The healthcare systems in European Union countries performed satisfactorily during the pandemic [3]. However, smaller countries coped more efficiently than larger countries, with significant variations being attributed to the wealth level of the countries included in the sample, whereas OECD countries were negatively affected in terms of healthcare efficiency from the pandemic’s onset [4].

Another strand of the literature examines the formation of public perceptions regarding healthcare system efficiency and related policy and goal-setting measures. Findings on public opinion formation have become increasingly important for designing policy on health service provision, resource planning, and target setting to better align with societal needs and improve patient satisfaction [5]. Moreover, understanding public attitudes and how they are formed is crucial for policymakers to ensure that proposed health measures are feasible and are communicated in ways that enhance acceptance [6]. In general, studies indicate that public perceptions about healthcare system efficiency have improved over the years, and these stances are largely affected by demographic and personal characteristics [7]. Studies also emphasize on the need to understand the pathways through which public opinion regarding healthcare is affected, not only for policy-making purposes, but also for restoring institutional trust and responsiveness to public guidelines during periods of crises [8]. Indeed, public trust in the healthcare system underpins the effectiveness of national healthcare systems, promotes public health, and improves societal well-being [9].

In light of the COVID-19 pandemic, researchers have focused on how public perceptions of healthcare systems were reshaped by the substantial social, economic, and well-being disruptions it caused. Public attitudes toward healthcare were greatly affected by the pandemic. However, a UK survey indicates that the public was more compliant and less critical of the national health system at the start of the pandemic; over time, as the pandemic unfolded, expectations and attitudes largely returned to pre-pandemic levels [10]. Other research detects evidence of a shift in public opinion in Colombia regarding national healthcare system performance during the COVID period, partly driven by individual healthcare needs and demand for healthcare services [11].

The existing literature underscores that public assessments of healthcare systems are shaped by a wide range of macroeconomic-level features and institutional characteristics, such as income levels, healthcare expenditure, and the accessibility features of the system [12]. Similarly—especially during the pandemic—trust in healthcare professionals significantly enhanced patient cooperation, responsiveness, and clinical health outcomes [13].

However, individual-level characteristics, such as socioeconomic status and demographic traits, remain under-investigated in the empirical literature, despite previous studies that highlight their pivotal role in shaping individual attitudes [14]. Most related studies examined how social preferences and attitudes (e.g., generosity and compliance with measures) were formed during the pandemic, while others investigated the evolution of institutional trust [15]. To the author’s knowledge, no study focuses specifically on the formation of public opinion about healthcare system performance before and after the pandemic. A study somewhat similar to the present one examined changes in public preferences regarding the implementation of a European health policy between 2020 and 2021 across countries in the European Union and similarly emphasizes the importance of demographic, socioeconomic, and occupational characteristics [16]. While that study shares some methodological features with the present one (it examines public stances in 2020 and 2021), the outcome differs, since it assesses individual positions on a European health policy rather than general perceptions of national healthcare system performance. Moreover, it is limited to countries in the European Union, and does not apply decomposition techniques to assess differences between 2020 and 2021.

This paper contributes to the literature by empirically examining how public perceptions of healthcare efficiency changed between pre- and post-COVID-19 periods in a large cross-country sample. Logistic regression models and Oaxaca decomposition analyses are applied within three country groups defined by similar geopolitical and economic characteristics (e.g., “Anglo World” group, “Europe” group, “East Asia” group). The methodological framework adopted aims at identifying the main determinants and sources of change in public assessments over time and to quantify their relative contributions to shifts in public attitudes. Disaggregating the sample into three groups of countries—a common approach in comparable research—improves interpretability and statistical precision by grouping countries with similar characteristics.

This study’s primary research questions, given sample availability, are as follows:

- What are the individual demographic, socioeconomic, and health-related determinants of individual evaluations of national healthcare system efficiency?
- Do the public’s assessments of healthcare differ between the pre-pandemic period (2011–2013) and the post-onset period (2021–2023)?
- Is there a gap in individual assessments of healthcare efficiency between these two periods?
- Can this gap be decomposed into observed and the unobserved components, and what is the contribution of each independent indicator to the observed gap?

Secondarily, clustering countries facilitates the detection of differences in respondents’ perceptions across groups with distinct institutional, economic, and social settings, thereby clarifying regional variation.

The next section (Section 2) presents the dataset and the empirical modelling methodology. Section 3 discusses the empirical findings. Finally, Section 4 concludes the paper.

## 2. Materials and Methods

### 2.1. Dataset

The data for the 18 countries are drawn from the International Social Survey Programme: Health and Health Care, which provides comparable information for the selected countries both before (2011–2013) and after the COVID-19 pandemic (2021–2023) (ISSP Research Group (2024): International Social Survey Programme: Health and Health Care I-II–Cumulation. GESIS, Cologne. Z8794 Data file Version 1.0.0, http://dx.doi.org/10.4232/1.14438 (accessed on 25 May 2025)). The dataset is a pooled cross-sectional one—the same individuals were not interviewed in both waves—but the national samples are drawn to be representative of their populations. The econometric models are estimated using the full country sample as well as three country sub-samples. The Country Similarity Index (https://objectivelists.com/country-similarity-index/ (accessed on 25 May 2025)) is employed to facilitate the clustering of countries into three groups based on a broad range of demographic, economic, political, cultural, and geographical characteristics: (1) Anglo World: Australia, United States (*n* = 5018). (2) Europe: Croatia, Czech Republic, Denmark, Finland, France, Germany, Italy, Netherlands, Norway, Poland, Slovakia, Slovenia, Switzerland (*n* = 34,897). (3) East Asia: China, Japan, Taiwan (*n* = 13,585). The dataset offers rich information on individual demographic, socioeconomic, and health-related characteristics. Given that the central research objective is to explore public perceptions of the efficiency of national healthcare systems before and after the pandemic, the dependent variable is derived from the following survey question: “How much do you agree or disagree with the following statement? ‘In general, the health care system in [country] is inefficient’”.

A binary (dummy) variable is constructed to distinguish between respondents who “strongly agree/agree” and those who “neither agree nor disagree/disagree/strongly disagree”.

The empirical models incorporate several individual-level variables, including demographic (age, gender, marital status), socioeconomic (education level, employment status, economic class), and health status indicators. In addition, respondents’ attitudes toward key features of the national healthcare system—such as their willingness to pay for healthcare system improvements and trust in doctors—are included as covariates.

Despite these controls, some unobserved factors remain unaccounted for because the survey waves are separated by a long interval and countries differ in their healthcare, economic, cultural, and social characteristics. To partly mitigate unobserved heterogeneity from group clustering and the long time gap, two annual macroeconomic indicators are included in the regression models. The first indicator is “Health expenditure as % of GDP”, drawn from the Global Health Expenditure Database from the World Health Organization. The second indicator is “Potential Years of Life Lost per 100,000 persons” drawn from the OECD Health Statistics Database. Both measures are crude indicators of national healthcare system performance and thus capture some country-level unobserved heterogeneity that could influence the results. However, in the extended models that include macroeconomic indicators, China and Taiwan are excluded due to missing data; therefore, the models cannot be estimated for the third country sample (East Asia).

Details on all variables used in the empirical analysis are provided in Table 1.

### 2.2. Methodology

Broadly speaking, the aim of this study is two-fold. First, it assesses differences in individuals’ views of national healthcare system efficiency between the pre-pandemic and post-pandemic periods. The Z-test on the equality of means is used to assess the equality of mean responses regarding healthcare efficiency between the pre-pandemic (2011–2013) and post-pandemic (2021–2023) groups. The test is statistically significant at the 1% level, indicating that there exists a difference in public assessments of healthcare system efficiency before and after 2020, the onset of the COVID-19 pandemic.

The dependent variable of interest is a binary variable that reflects respondents’ evaluations of whether the national healthcare system is inefficient. Consequently, logistic models with heteroskedasticity-robust standard errors are estimated. These models are applied separately to each time period for the full sample of countries, as well as for the three country clusters.

Second, this study investigates whether a statistically significant gap exists in assessments of healthcare efficiency before and after the pandemic, and quantifies the relative contribution of each explanatory factor to this gap. To achieve this, the standard Blinder-Oaxaca decomposition method, commonly applied in similar research [17,18,19], is employed. This study adopts a nonlinear extension of the model [20].

In this context, the decomposition of individual healthcare efficiency assessments (denoted as the dependent variable *Y*) between the two time periods (2011–2013 (*period* A) vs. 2021–2023 (*period B*)) is expressed as follows:

(1) $${\bar{Y}}_{period\;A}-{\bar{Y}}_{period\;B}=\bar{F\left(X_{period\;A}\beta_{period\;A}\right)}-\bar{F\left(X_{period\;B}\beta_{period\;B}\right)}=\;\left(\bar{F\left(X_{period\;A}\beta_{period\;A}\right)}-\bar{F\left(X_{period\;B}\beta_{period\;A}\right)}\right)+\left(\bar{F\left(X_{period\;B}\beta_{period\;A}\right)}-\bar{F\left(X_{period\;B}\beta_{period\;B}\right)}\right)$$

Equation (1) decomposes the difference in average assessments of healthcare efficiency into two main components. The first term, $\left(\bar{F\left(X_{period\;A}\beta_{period\;A}\right)}-\bar{F\left(X_{period\;B}\beta_{period\;A}\right)}\right)$, represents the explained component or characteristics effect. This portion of the gap is attributable to differences in observed characteristics (i.e., covariates) between the two periods. The explained component captures the expected difference in average healthcare efficiency assessments if the 2011–2013 respondents had the same distribution of characteristics as those in 2021–2023 [20]. A positive coefficient for a specific covariate in the explained component suggests that equalizing the distribution of that variable across periods would reduce the efficiency perception gap [21,22].

The second term, $\left(\bar{F\left(X_{period\;B}\beta_{period\;A}\right)}-\bar{F\left(X_{period\;B}\beta_{period\;B}\right)}\right)$, captures the unexplained component or coefficients effect. This part reflects changes in behavioural or cultural responses to the same characteristics across the two time periods. A negative coefficient indicates an increase in the efficiency perception gap attributable to unobserved factors; essentially, it measures how perceptions would differ if individuals in 2011–2013 responded to the predictors in the same way as those in 2021–2023 [20,21,23].

Both linear and nonlinear decomposition methods are frequently used to disentangle the contributions of observed and unobserved factors to differences in outcomes. However, these models are sensitive to functional form, model specification, and potential misspecification biases [20,24]. The nonlinear approach adopted here effectively addresses several known biases, namely the identification bias (i.e., the sensitivity to the selection of reference groups for categorical variables), the indexing bias (i.e., the sensitivity to the choice of comparison and reference groups) and the path dependence problem (i.e., sensitivity to the order in which variables are included in the model). These concerns are addressed using the methodological refinements proposed by relevant studies [20,22,25,26,27].

## 3. Results

### 3.1. Descriptive Statistics

Table 2 presents the mean values of the variables included in this study, disaggregated by time period (between 2011–2013 and 2021–2023). The main variable of interest is the index capturing individual assessments of the inefficiency of particular national healthcare systems. As shown in Table 2, perceptions of healthcare inefficiency appear to have improved in the post-pandemic period. While approximately 35.9% of respondents rated their national healthcare system as inefficient in 2011–2013, this figure declined to 27.9% in 2021–2023.

In both periods, the majority of respondents are middle-aged (49–52 years), female, and married. The educational profile of the sample has improved over time. Respondents in the 2021–2023 sample report higher levels of educational attainment but slightly lower levels of employment, and income compared to those in the 2011–2013 sample.

Similarly, trust in doctors is slightly higher in the later period, with 78.4% of respondents expressing trust in 2021–2023 compared to 73.7% in 2011–2013. Willingness to finance the national health system through increased taxation also remains relatively stable across periods, i.e., 33.3% in 2021–2023 versus 31.9% in 2011–2013. Self-reported health status has improved. In the 2021–2023 group, 72.4% of respondents report higher levels of health, compared to 67.7% in the earlier period. Average health expenditure rose from 8.625% of GDP to 10.249% of GDP, possibly reflecting the higher economic costs of responding to the pandemic. On the other hand, the indicator of premature mortality, namely Potential Years of Life Lost, seems to be slightly lower on average in the 2021–2023 period. Studies also report major discrepancies between countries, and attribute decreases in premature mortality over the years to increased COVID-19 mortality among older adults (yielding fewer years lost per death), concurrent declines in some causes of premature death, and pandemic-related reductions in injuries that lowered younger-age mortality [28,29,30,31].

### 3.2. Logistic Regression Results

The logistic regression results, disaggregated by time period, are presented in Table 3, Table 4, Table 5 and Table 6 for the total sample and the three country groups. The estimations are augmented with macroeconomic indicators as a robustness check of the original models. All regression estimates are reported as odds ratios, and the model fit is satisfactory. Overall, the augmented specifications do not substantially alter the results.

Table 3 presents the regression estimates for the full sample of countries. Notable differences emerge between the two time periods. In the pre-COVID era, key determinants of individual assessments of national healthcare efficiency include gender, educational status, trust in doctors, and self-reported health status. In contrast, during the post-COVID era, the significant determinants are gender, marital status, employment status, trust in doctors, and health status, with the latter two remaining consistently influential across both periods. The same finding is observed for the two macroeconomic indicators, namely health expenditure and premature mortality, which seem to consistently exert an effect upon individual healthcare assessments.

More specifically, during 2011–2013, higher educational attainment is associated with an increased likelihood of evaluating the healthcare system as inefficient: individuals with high educational status have 11.6% higher odds and those with middle educational status have 9.7% higher odds to assess the system as inefficient compared to those with low educational status. This suggests that individuals with higher educational backgrounds tend to evaluate the performance of national health systems more critically. In the augmented models, the effect of educational status disappears. Respondents with higher trust in doctors have 50.3% lower odds of assessing healthcare as inefficient, and respondents in good health have 16.2% lower odds. These estimates change only slightly when macroeconomic indices are included. Higher health expenditure is associated with 29.6% lower odds of judging healthcare as inefficient. Although statistically significant, Potential Years of Life Lost has only a small quantitative effect on individual assessments of the national healthcare system.

In the 2021–2023 period, demographic characteristics appear to play a stronger role in shaping individual assessments. Males have 18.7% higher odds than females to consider the healthcare system as inefficient. Similarly, married respondents have 9.1% higher odds of expressing negative evaluations compared to their unmarried counterparts.

Employment status also plays a significant role. Being employed is associated with 12.7% lower odds of perceiving the system as inefficient, while individuals outside the labour force show 21% lower odds, in comparison to the unemployed. These effects attenuate when macroeconomic indicators are introduced in the models. Once again, higher trust in doctors and better health status are linked to lower odds (53.5% and 19.8%, respectively) of reporting inefficiency in the national healthcare system. This suggests that respondents with better health status, and thus potentially lower reliance on health services, tend to be more adaptable or tolerant toward the performance of the healthcare system. An increase in national health expenditure is associated with 12.6% lower odds of assessing the healthcare system as inefficient. The quantitative impact of the years of life lost is also negligible in the 2021–2023 period.

Roughly speaking, across all three country groups, a consistent pattern mirrors the full-sample findings: individuals in a better health state and with greater trust in doctors tend to view their national healthcare system performance more favourably. A similar protective effect on individual assessments is observed when health expenditure increases.

Table 4 presents the corresponding regression results for the Anglo-world country group (i.e., Australia and the United States). In this subgroup, individual socioeconomic characteristics do not appear to significantly influence perceptions of healthcare system efficiency either before or after the onset of the pandemic.

In the 2011–2013 period, increased age is associated with slightly higher odds of perceiving the healthcare system as inefficient; however, the quantitative effect is marginal. Interestingly, respondents with medium educational attainment exhibit 21.4% lower odds of evaluating the healthcare system as inefficient compared to those with low educational status. Consistent with previous findings, higher trust in doctors is associated with substantially lower odds of perceiving inefficiency (i.e., 44.3% lower in the pre-COVID period and 36.7% lower in the post-COVID period). Likewise, respondents in better health report lower odds of negative assessments (20.7% lower before the pandemic and 26.2% lower after).

Compared to the full sample, willingness to finance the healthcare system through taxation has a significant impact upon individual stances to healthcare. In both periods, those expressing such willingness are more likely to view the system as inefficient (36.6% higher odds pre-COVID and 48.7% higher odds post-COVID). This may reflect greater expectations and the awareness of system limitations among individuals willing to contribute financially, particularly after the onset of COVID. Surprisingly, including macroeconomic indicators does not change the initial estimates. An increase in health expenditure is associated with 8.9% higher odds of assessing the healthcare system as inefficient for 2011–2013, and 22.7% higher odds for 2021–2023. While these estimates diverge from the total sample, studies indicate that US citizens often view their high-spending health system as inefficient, attributing dissatisfaction to high costs, insurance complexity, and unequal access [32]. On the same note, Australian citizens tend to favourably assess their national healthcare, but are concerned about rising healthcare costs [7,33].

Taken together, these findings suggest that health- and healthcare-related variables are stronger drivers of individual stances than socioeconomic characteristics: in particular, individual health status, trust in doctors, and health expenditure appear to be central to shaping evaluations of healthcare system performance in Anglo-world countries.

Table 5 presents the logistic regression results for the European country sample, which includes Croatia, Czech Republic, Denmark, Finland, France, Germany, Italy, Netherlands, Norway, Poland, Slovakia, Slovenia, and Switzerland.

In this group, alongside consistent effects of health- and healthcare-related variables, gender, marital status, and employment status significantly influenced perceptions of healthcare system efficiency, but only for 2021–2023. Specifically, males had 24% higher odds of rating healthcare as inefficient; married respondents had 9.4% higher odds; and employed respondents and those out of the labour force had 20.7% and 28.8% lower odds, respectively, of rating healthcare as inefficient compared to unemployed respondents.

As in other country groups, health- and healthcare-related characteristics remain the most significant drivers of individual assessments, regardless of the time period. Higher trust in doctors is strongly associated with lower odds of negative evaluations (55.3% lower in the pre-pandemic period and 57% lower in the post-pandemic period), highlighting the role of institutional trust in shaping healthcare attitudes. Similarly, better self-reported health status corresponds to 14.5% lower odds of perceiving the system as inefficient in 2011–2013 and 21.1% lower odds in 2021–2023. Additionally, willingness to pay higher taxes for healthcare is linked to 10.2% lower odds of negative evaluations, but this effect is statistically significant only in the post-COVID period. As is the case in the Anglo-world sample, including macroeconomic indicators does not alter the estimates; however, health expenditure and years of life lost are statistically insignificant in both waves.

Overall, these findings suggest that, as in the aforementioned country groups, individual health status and doctor trust are central to shaping public assessments of national healthcare system performance across Europe. Notably, the respective effects appear to be slightly more pronounced during the post-COVID period, suggesting that the experience of the pandemic might have played a role in shaping and intensifying these individual assessments.

Table 6 presents the regression results for the East Asia sample, which includes China, Japan, and Taiwan. As noted above, the macroeconomic indices could not be controlled for in the regressions for the East Asia sample due to missing data. A similar pattern to the other country groups is observed, confirming the consistency of the findings. In this region, age, gender, and educational status influence individual assessments of healthcare efficiency, but only in the pre-COVID period.

More specifically, in the 2011–2013 period, higher age is associated with slightly lower odds of perceiving the system as inefficient; however, the effect is statistically significant, but negligible in magnitude. Male respondents show 12% higher odds of evaluating the healthcare system as inefficient. Similarly, individuals of middle and high educational status exhibit 22.6% and 24.7% higher odds, respectively, of reporting inefficiency compared to those of lower educational status. These findings suggest that more educated respondents may hold higher performance expectations.

In the 2021–2023 period, individual demographic, socioeconomic, and occupational characteristics do not appear to influence the formation of healthcare stances. Once again, trust in doctors plays a central role. Higher doctor trust is associated with significantly lower odds of perceiving inefficiency (40.6% lower in the pre-COVID period and 48.6% lower in the post-COVID period). Similarly, higher self-reported health status is associated with 17.5% lower odds of assessing the system as inefficient in 2011–2013 and 14.8% lower odds in 2021–2023. Finally, willingness to pay higher taxes for healthcare is associated with 12% lower odds of negative assessments, although this effect is only detected in the pre-COVID period.

These findings for the East Asia sample reinforce the broader conclusion that individual health status and trust in health institutions are persistent and powerful predictors of public perceptions of healthcare system efficiency. Moreover, the emergence of different determinants across time periods suggests that the pandemic has shaped the relative influence of demographic and socioeconomic factors on these assessments in the East Asian context.

### 3.3. Oaxaca Decomposition Results

The Oaxaca decomposition models offer valuable insights into the underlying drivers of individual assessments of national healthcare system efficiency. Specifically, these models help to disentangle the factors that either widen or reduce the gap in perceived healthcare inefficiency between the two periods under study.

Table 7 presents the results of the decomposition analysis for all countries. The models quantify the contribution of both explained (observable) and unexplained (unobservable) components to the gap in healthcare assessments between 2011–2013 and 2021–2023.

On average, there is an 8% gap in healthcare efficiency assessments, indicating that more respondents rated their national healthcare systems as inefficient during 2011–2013 compared to the more recent period of 2021–2023. The gap is reduced to 6.1% when macroeconomic indicators are controlled for in the estimations.

The decomposition results for the baseline models show that this gap is primarily driven by differences in coefficients, which account for approximately 92% of the total gap. This suggests that behavioural, cultural, and institutional factors (which reflect the ways individuals interpret and respond to the circumstances) are the dominant contributors to the observed differences in assessments over time. However, including macroeconomic indicators reverses the decomposition results: the explained component accounts for 54.79% of the gap, while the unexplained component accounts for 45.21%. This shift indicates that incorporating differences in countries’ healthcare settings both reduces the gap and helps explain part of the change in individual stances over time. The discussion will therefore focus on the augmented model’s findings. While the relative contribution of individual characteristics remains largely unchanged after controlling for country-level healthcare variables, those country-level indices still explain a substantial share of the gap in respondents’ assessments.

Turning to the explained component of the assessment gap, changes in the distribution of observable characteristics (age, gender, education, employment status) explain only a small share of the gap. The first two columns in the lower-right panel of estimates of Table 7 presents each factor’s percentage contribution to the overall gap in healthcare assessments. Equalizing levels of doctor trust across the two time periods would be expected to reduce the gap by 13.14%, assuming that respondents in the pre-COVID period held similar levels of trust as those in the post-COVID period. This finding is consistent with expectations, as individuals in 2011–2013 expressed more negative assessments of their national healthcare systems and lower doctor trust compared to those in 2021–2023. A similar, although smaller, effect is observed for health status: equalizing health status evaluations would reduce the gap by 0.10%. Equalizing healthcare expenditure between the two periods would reduce the gap by 12.59%, and equalizing potential years of life lost would reduce the gap by 45.90%. These results suggest that lower doctor trust, lower health expenditure, and higher premature mortality in the earlier period contributed to the disparity in perceptions of healthcare efficiency between the two periods.

In contrast, equalizing educational attainment would be expected to increase the observed gap. When considered alongside the summary statistics in Table 2, this finding implies that if individuals in 2011–2013 had possessed the higher educational levels observed in 2021–2023, their evaluations of the healthcare system would have been even more critical, thereby widening the gap between the two periods. Other statistically significant contributions are detected; however, their magnitude is negligible and unlikely to meaningfully impact the overall gap in healthcare assessments.

The lower-left panel of Table 7 shows the percentage contribution of unobserved characteristics (differences in responses to covariates) to the gap. The unobserved component is mainly driven by differential reactions to demographic characteristics. Aligning responses by educational status would substantially reduce the gap (by 53.51% for higher education and 79.20% for middle education). If employed respondents had similar perceptions across periods, the gap would shrink by 53.03%; a comparable alignment for those out of the labour force would reduce the gap by 27.96%. Equalizing reactions by gender, however, would increase the gap by 19.37%. These results indicate that differential behavioural responses by gender, education, and employment status are major contributors to the divergence in healthcare system evaluations between the pre- and post-COVID periods. Equalizing reactions on willingness to pay would be expected to reduce the gap in healthcare assessments, suggesting that changes in perceptions about personal contributions to healthcare funding—especially under COVID-related pressures—have tended to reinforce rather than narrow the divide.

Table 8 reports the decomposition results for the Anglo-world group. The unexplained component overwhelmingly drives the gap (166.63%), while the explained component is not statistically significant, implying that changes in respondents’ reactions, not changes in their characteristics, primarily account for the assessment differences between waves. Differences in the distribution of characteristics (age, education, doctor trust, health status, willingness to pay) have only minor effects (0.94–3.46%). An exception is healthcare expenditure: namely, equalizing expenditure across waves would increase the gap by 62.04%, indicating that, if higher expenditure had occurred in 2011–2013, first-wave respondents would likely have been even more negative in their assessments. Similarly, differential behavioural responses to healthcare expenditure have a very large impact, since equalizing those responses would raise the gap by roughly 505%. This suggests that increases in health expenditure weighed less negatively on respondents’ assessments in 2021–2023 than they would have in 2011–2013, possibly reflecting greater tolerance for higher expenditure under pandemic conditions.

Table 9 presents the results of the decomposition analysis for the European country group, revealing a pattern similar to that observed in the Anglo-world group. The contribution of the observed component is substantial, amounting to 162.86%, which more than offsets the contribution of the unobserved component (−62.86%).

Although some differences in respondent characteristics across the two time periods are found to influence the gap, their quantitative impact is quite low. The most notable contribution arises from doctor trust and healthcare expenditure. Specifically, equalizing the levels of doctor trust between the 2011–2013 and 2021–2023 periods would reduce the gap in healthcare system assessments by 16.41%. Given that doctor trust was higher in 2021–2023, this suggests that the lower trust levels observed during the earlier period played a significant role in shaping more negative evaluations of national healthcare systems. Equalizing healthcare expenditure between the two periods would decrease the gap by 173.57%. Unexpectedly, expenditures were slightly lower in 2011–2013. This means that increasing spending in that period would have narrowed the public opinion gap on healthcare system performance.

Turning to the contribution of the unobserved factors to the gap, only a few significant findings emerge for the European sample; however, their quantitative impact is quite considerable. Equalizing the behavioural responses of respondents with higher and middle educational status across the two periods would reduce the healthcare assessment gap by 58.29% and 74.09%, respectively. In contrast, equalizing the responses of male respondents would actually increase the gap by 24.21%, suggesting that shifts in how men evaluated the healthcare system before and after the pandemic may have contributed to narrowing the observed difference over time. The most prominent effect is observed once again for healthcare expenditure. Equalizing responses to expenditure between the two waves would increase the gap by about 606.97%. Similarly, equalizing responses regarding premature mortality (namely, potential years of life lost) would increase the gap by 212.94%.

Once again, a similar pattern emerges for the East Asia sample. Unfortunately, the macroeconomic indicators of healthcare expenditure and premature mortality cannot be utilized for this group due to missing data. The majority of the gap in healthcare system assessments is attributed to unobserved characteristics (94.25%), while the observed characteristics contribute only marginally (5.75%). The results are presented in Table 10.

The effects of differences in the distribution of observed covariates are relatively minor, ranging from 0.34% to 6.19%. Specifically, equalizing doctor trust between the two time periods would reduce the gap by 6.19%. Likewise, if respondents in the 2011–2013 sample had similar higher educational class distribution to those in the 2021–2023 sample, the gap would be expected to increase by 5.19%. A similar age distribution would decrease the gap by 2.77%.

Turning to unobserved characteristics, differences in behavioural responses to high income account for a notable portion of the gap. Equalizing these responses would reduce the gap by 9.06% for respondents with a higher income status. Equalizing the responses of the sample regarding doctor trust between the two waves would decrease the gap by 30.48%.

## 4. Discussion and Conclusion

This study examines individual perceptions of national healthcare system efficiency before and after the COVID-19 pandemic across 18 countries, grouped into three distinct cultural and geopolitical clusters: the Anglo-world, Europe, and East Asia. Using individual-level data from the International Social Survey Programme (ISSP) for the periods 2011–2013 and 2021–2023, this analysis investigates the demographic, socioeconomic, and health-related factors that shape public attitudes toward healthcare systems. In addition, macroeconomic healthcare indicators are controlled for in this analysis. Both Logistic regression and Oaxaca–Blinder decomposition techniques are employed to identify not only the significant drivers of these perceptions, but also the sources of change over time.

The results indicate that health-related factors, particularly doctor trust and self-assessed health status, consistently drive perceptions of healthcare efficiency across all time periods and country groups. Individuals with better health and greater trust in doctors are significantly less likely to evaluate their national healthcare systems as inefficient. To the author’s knowledge, no prior studies have examined exactly the same research questions. However, empirical evidence links institutional trust—particularly trust in the healthcare workforce—with higher compliance with health guidelines and more positive attitudes toward communicated information [34]. Institutional trust and good health also positively influence perceptions of healthcare efficiency in Germany [35]. More broadly, poorer health and lower institutional trust have repeatedly been associated with stricter, more critical stances toward the healthcare system [36,37]. Personal perceptions and experiences, access to services, and ideological positions likely drive these patterns, since individuals in better health are less reliant on the system and thus tend to hold more favourable views, while those with higher trust in healthcare evaluate their experiences more positively, yielding more generous assessments [36,38,39]. Increased healthcare expenditure is also associated with more favourable public views of healthcare, with quantitatively substantial effects [36]. Higher spending appears to have a protective effect on public attitudes, as perhaps it is perceived as an investment or improvement in the healthcare system.

Demographic and socioeconomic factors—gender, age, marital and employment status, and willingness to pay for better care—also shape attitudes toward the healthcare system, but their effects vary by region and period and are generally less consistent than health-related determinants. Overall, higher educational attainment is associated with more critical views of national healthcare inefficiencies [36], while labour-market participation tends to correlate with less critical assessments. Men, married respondents, and older individuals have higher odds of judging the system as inefficient. Previous studies also found weak evidence that younger people and women are less likely to express positive assessments, and that positive evaluations have risen in more recent years [7]. Other studies also highlight the role of health status, healthcare needs, and economic conditions in shaping public sentiment, broadly supporting the present study’s findings [6,16].

All in all, the logistic regression results indicate that health-related factors—individual health status, trust in healthcare providers, and total healthcare expenditure—are the primary determinants of public assessments of healthcare systems. Demographic and socioeconomic characteristics (gender, age, marital status, educational and employment status, willingness to pay) show smaller, less consistent effects, and are sensitive to specification and regional variation. Importantly, the set of dominant predictors remains stable before and after the onset of COVID. Despite increased pressure on national systems after 2020, health factors continue to explain most variation in evaluations across regions and periods. These findings imply that policies improving health outcomes, service provision, and institutional trust are more likely to shift public opinion than interventions targeted solely at demographic or socioeconomic groups.

Although the logistic regressions are robust to alternative specifications, the decomposition analysis highlights healthcare expenditure as the key driver of the gap in individual assessments between 2011–2013 and 2021–2023. Overall, respondents in 2021–2023 report more favourable views of their healthcare systems than those in 2011–2013, a pattern consistent with the pandemic “rally-round-the-flag” documented in the literature [40]. However, prior studies also indicate this boost in support is largely short-lived, with public sentiment tending to revert toward pre-COVID attitudes as the crisis endures [10,41,42].

The decomposition of the assessment gap between 2011–2013 and 2021–2023 shows that the post-pandemic improvement in healthcare evaluations reflects both changes in observed characteristics and in unobserved behavioural or institutional shifts. Across all groups, healthcare expenditure is the single most influential factor, followed by trust in doctors. In detail, differences in the distribution of these variables between waves account for much of the gap. Demographic (gender), educational, and employment variables also contribute, but primarily through changing responses. Respondents with higher education and those in employment altered their reactions across waves, thereby partly driving the shift in attitudes toward the healthcare system.

The Anglo-world and East Asia samples show particularly strong effects from unobserved characteristics, highlighting the influence of cultural, institutional, or pandemic-driven shifts in public sentiment. By contrast, the European sample displays smaller explained contributions overall, although key covariates (namely, healthcare expenditure, trust in doctors, and educational and employment status) still meaningfully reduce the assessment gap. Across all regions, national healthcare expenditure consistently contributes to the gap. Both differences in healthcare spending distribution and differences in responses to it help explain changing assessments. This pattern suggests that expenditure shapes public opinion by improving perceived service quality, access, and equity and by moderating public reactions to system performance [43,44]. Policymakers should therefore consider how spending decisions influence public attitudes when designing healthcare reforms.

Unobserved behavioural, institutional, and cultural factors appear to shape public opinion on healthcare over time. The findings of this study suggest that even individuals with similar health, healthcare, and socioeconomic profiles often report different evaluations of their national systems, reflecting divergent beliefs and responses. Although these factors are difficult to measure and are therefore not fully controllable in the regression models, prior work links ideology, cultural values, welfare-state context, and perceptions of fairness to healthcare evaluations. For instance, more egalitarian or anti-hierarchical cultures tend to produce more positive assessments [36], political ideology mediates citizens’ evaluations of system performance [16], and generous welfare states foster greater support for healthcare investment via reinforcing feedback mechanisms. Individual experiences from national healthcare systems, such as barriers to access, also strongly influence attitudes [35,37]. Given the data constraints, these psychological and cultural pathways cannot be directly tested in this study, but the literature suggests that they plausibly contribute to the patterns observed here. Based on the above-mentioned findings, policymakers should invest in visible service improvements and targeted spending while simultaneously building trust through transparency, community engagement, and research on cultural and behavioural drivers so that increased resources translate into fairer and more accessible care that, in turn, leads to sustained public confidence.

There are some limitations that should be acknowledged, and the findings should be viewed with caution. First, this study examines the gap in healthcare assessments across three country groups over roughly a ten-year period. During this decade, many factors, such as policy and institutional reforms, continuously shaped assessments, so unobserved heterogeneity over time and across different country contexts may drive some results. The inclusion of year and country dummies, together with national healthcare indicators (namely, healthcare expenditure and Potential Years of Life Lost), aims to limit unobserved heterogeneity while capturing differential national settings. In addition, the assessment gap detected in this study is consistent with the literature documenting increased support for incumbents and institutions in the early pandemic (“rally-round-the-flag” effects), so it is unlikely to be entirely driven by unobserved heterogeneity. Finally, the finding that the factors driving public assessments are similar across the two periods (primarily health- and healthcare-related variables) probably limits the role of intermediate unknown pathways over the ten-year interval. Notwithstanding these limitations, this study sheds light on public assessments of healthcare across time and a wide range of countries, a topic that is under-researched, to the author’s knowledge. Future research should aim to disentangle the cultural, behavioural, and institutional pathways that mediate citizens’ reactions to national healthcare systems, independently of socioeconomic position.

Overall, the findings underscore the importance of national healthcare spending, public trust in medical professionals, perceived personal health, and the broader behavioural context in shaping how individuals evaluate the performance of healthcare systems. Those factors are found to be the primary determinants of public assessments of healthcare systems, more so than demographic or socioeconomic traits. National healthcare expenditure substantially shapes public opinion, both through its distribution and via how citizens respond to spending. Unobserved cultural, behavioural, and institutional factors meaningfully influence evaluations and can produce divergent views among similar individuals, so attention to national settings and perceptions is crucial. As such, policy actions aiming at investments and efficient resource allocation, strengthening doctor–patient relationships, adequately addressing overall population health needs, and addressing cultural and behavioural drivers will not only enhance the healthcare system performance, but also will increase the public’s satisfaction with and perceived efficiency of the healthcare system, particularly during times of health system stress, such as the recent pandemic experience. Gaining the support of citizens for health policies is crucial, since it strengthens trust in national authorities and increases compliance with measures needed to protect and further promote public health [45].

## Figures and Tables

**Table 1:** Definition of variables.

Variable Names	Definition
**Dependent variable**	
Healthcare efficiency	1: Respondent “strongly agrees/agrees” that the healthcare system in own country is inefficient; 0: Otherwise
**Independent variables**	
Age	Age in years (15–95 years of age)
Males	1: Respondent is male; 0: Otherwise
Married	1: Respondent is married/living with a partner; 0: Otherwise
High educational status	1: Respondent has completed levels 5–8 based on ISCED 2011 classification; 0: Otherwise
Medium educational status	1: Respondent has completed levels 3–4 based on ISCED 2011 classification; 0: Otherwise
Low educational status	1: Respondent has completed levels 0–2 based on ISCED 2011 classification; 0: Otherwise (omitted from regressions)
Employed	1: Respondent is employed; 0: Otherwise
Unemployed	1: Respondent is unemployed; 0: Otherwise (omitted from regressions)
Out of labour force	1: Respondent is out of labour force (e.g., in education, health problems, retired, performing housework, etc.); 0: Otherwise
High income	1: Respondent belongs in the “high income” category; 0: Otherwise
Middle income	1: Respondent belongs in the “middle income” category; 0: Otherwise
Low income	1: Respondent belongs in the “low income” category; 0: Otherwise (omitted from regressions)
Willingness to pay for healthcare	1: Respondent is “very/fairly” willing to pay higher taxes for national healthcare system improvement; 0: Otherwise
Doctor trust	1: Respondent “strongly agrees/agrees” that, all things considered, doctors can be trusted; 0: Otherwise
Self-assessed health status	1: Respondents argues that he/she is in “excellent/very good/good” health”; 0: Otherwise
Health expenditure (% of GDP)	Level of current health expenditure expressed as a percentage of GDP
Potential Years of Life Lost (per 100,000 persons)	A summary measure of premature mortality occurring at each age which are, a priori, preventable

Notes: Individual-level data are drawn from the ISSP Research Group (2024): International Social Survey Programme: Health and Health Care I-II—Cumulation, GESIS, Cologne. Health expenditure (% of GDP) data are drawn from the Global Health Expenditure Database, World Health Organization. Potential Years of Life Lost (per 100,000 persons) information is drawn from the OECD Health Statistics Database.

**Table 2:** Descriptive statistics, mean values.

Variable Names	2011–2013	2021–2023
Healthcare efficiency	0.359 ***	0.279 ***
Age	48.852	51.689
Males	0.470	0.463
Married	0.617	0.569
High educational status	0.276	0.399
Medium educational status	0.366	0.406
Employed	0.582	0.576
Out of labour force	0.375	0.390
High income	0.271	0.266
Middle income	0.268	0.269
Willingness to pay for healthcare	0.319	0.333
Doctor trust	0.737	0.784
Self-assessed health status	0.677	0.724
Health expenditure (% of GDP)	8.625	10.249
Potential Years of Life Lost (per 100,000 persons)	4874.080	4324.218
Observations	29,301	24,199

Note: *** denotes statistical significance at 1% level in the *z*-test on the equality of means between the sub-samples. Observations are slightly lower for the 2011–2013 (*n* = 21,934) and 2021–2023 (*n* = 20,165) samples due to the exclusion of China and Taiwan.

**Table 3:** Determinants of individual perceptions about healthcare systems efficiency by year. Total sample. Logistic regression models.

	Dependent Variable	2011–2013		2021–2023	
Independent Variables		Total Sample Coefficients are Expressed in Odds Ratio			
Age		0.999	1.001	1.000	1.000
Males		1.056 **	1.040	1.187 ***	1.216 ***
Married		1.018	1.053	1.091***	1.108 ***
High educational status		1.116 **	1.044	0.985	1.014
Medium educational status		1.097 ***	1.023	0.967	0.961
Employed		1.085	1.077	0.873	0.790 ***
Out of labour force		0.986	0.950	0.790 ***	0.701 ***
High income		1.051	1.003	0.998	1.018
Middle income		1.006	0.950	0.992	0.996
Willingness to pay for healthcare		0.975	1.031	0.967	0.984
Doctor trust		0.497 ***	0.476 ***	0.465 ***	0.462 ***
Self-assessed health status		0.838 ***	0.856 ***	0.802 ***	0.795 ***
Health expenditure (% of GDP)			0.704 ***		0.874 ***
Potential Years of Life Lost (per 100,000 persons)			1.001 ***		1.000 ***
Constant		1.514 ***	0.174 ***	0.863	0.781
Country dummies		Yes	Yes	Yes	Yes
Pseudo R2		0.10	0.13	0.12	0.13
Wald chi squared		2798.16 ***	2663.00 ***	2818.22 ***	2587.43 ***
Observations		29,301	21,394	24,199	20,165

Note: Statistical significance is denoted by ** *p* < 0.05; *** *p* < 0.01. Regressions are estimated with heteroskedasticity-robust standard errors.

**Table 4:** Determinants of individual perceptions about healthcare systems efficiency by year. Anglo-world sample. Logistic regression models.

	Dependent Variable	2011–2013		2021–2023	
Independent Variables		Anglo-World Sample Coefficients are Expressed in Odds Ratio			
Age		1.005 **	1.005 **	1.003	1.003
Males		1.063	1.063	1.011	1.011
Married		0.946	0.946	1.190	1.190
High educational status		0.858	0.858	1.044	1.044
Medium educational status		0.786 **	0.786 **	0.850	0.850
Employed		1.331	1.331	0.789	0.789
Out of labour force		1.175	1.175	0.670	0.670
High income		1.102	1.102	1.173	1.173
Middle income		0.961	0.961	1.125	1.125
Willingness to pay for healthcare		1.366 ***	1.366 ***	1.487 ***	1.487 ***
Doctor trust		0.557 ***	0.557 ***	0.633 ***	0.633 ***
Self-assessed health status		0.793 ***	0.793 ***	0.738 ***	0.738 ***
Health expenditure (% of GDP)			1.089 ***		1.227 ***
Potential Years of Life Lost (per 100,000 persons)					
Constant		0.925	0.445 ***	0.554 *	0.062 ***
Country dummies		Yes	Yes	Yes	Yes
Pseudo R2		0.04	0.04	0.10	0.10
Wald chi squared		152.93 ***	152.93 ***	229.80 ***	229.80 ***
Observations		3134	3134	1884	1884

Note: Statistical significance is denoted by * *p* < 0.1; ** *p* < 0.05; *** *p* < 0.01. Regressions are estimated with heteroskedasticity-robust standard errors. Potential years of life lost are omitted from regressions due to collinearity.

**Table 5:** Determinants of individual perceptions about healthcare systems efficiency by year. European sample. Logistic regression models.

	Dependent Variable	2011–2013		2021–2023	
Independent Variables		European Sample Coefficients are Expressed in Odds Ratio			
Age		1.000	1.000	1.001	1.001
Males		1.029	1.029	1.240 ***	1.240 ***
Married		1.079	1.079	1.094 **	1.094 **
High educational status		1.044	1.044	0.981	0.981
Medium educational status		1.064	1.064	0.984	0.984
Employed		1.056	1.056	0.793 **	0.793 **
Out of labour force		0.865	0.865	0.712 ***	0.712 ***
High income		0.974	0.974	0.972	0.972
Middle income		0.935	0.935	1.001	1.001
Willingness to pay for healthcare		0.956	0.956	0.898 ***	0.898 ***
Doctor trust		0.447 ***	0.447 ***	0.430 ***	0.430 ***
Self-assessed health status		0.855 ***	0.855 ***	0.789 ***	0.789 ***
Health expenditure (% of GDP)			0.689		1.064
Potential Years of Life Lost (per 100,000 persons)			0.999		1.000
Constant		0.622 ***	82.258	1.158	0.115
Country dummies		Yes	Yes	Yes	Yes
Pseudo R2		0.13	0.13	0.14	0.14
Wald chi squared		2018.29 ***	2018.29 ***	2115.08 ***	2115.08 ***
Observations		17,792	17,792	17,105	17,105

Note: Statistical significance is denoted by ** *p* < 0.05; *** *p* < 0.01. Regressions are estimated with heteroskedasticity-robust standard errors.

**Table 6:** Determinants of individual perceptions about healthcare systems efficiency by year. East Asia sample. Logistic regression models.

	Dependent Variable	2011–2013	2021–2023
Independent Variables		East Asia Sample Coefficients are Expressed in Odds Ratio	
Age		0.995 ***	0.997
Males		1.120 **	1.109
Married		0.965	1.017
High educational status		1.247 ***	0.909
Medium educational status		1.226 ***	0.973
Employed		1.146	1.446
Out of labour force		1.197	1.362
High income		1.078	0.895
Middle income		1.086	0.908
Willingness to pay for healthcare		0.880 ***	0.956
Doctor trust		0.594 ***	0.514 ***
Self-assessed health status		0.825 ***	0.852 ***
Constant		1.019	0.406 ***
Country dummies		Yes	Yes
Pseudo R2		0.02	0.04
Wald chi squared		257.07 ***	232.13 ***
Observations		8375	5210

Note: Statistical significance is denoted by ** *p* < 0.05; *** *p* < 0.01. Regressions are estimated with heteroskedasticity-robust standard errors. The macroeconomic indicators are excluded from the estimations due to missing data for the two out of the three countries of the sample.

**Table 7:** Determinants of individual perceptions about healthcare systems efficiency. Total sample. Oaxaca decomposition.

	Groups	2011–2013 vs. 2021–2023			
Variable Names					
Gap in individual perceptions about healthcare systems efficiency between the two waves		0.080 ***	0.061 ***	0.080 ***	0.061 ***
Characteristics (explained difference)		0.006 *** (7.76%)	0.033 *** (54.79%)	0.006 *** (7.76%)	0.033 *** (54.79%)
Coefficients (unexplained difference)		0.074 *** (92.24%)	0.028 *** (45.21%)	0.074 *** (92.24%)	0.028 *** (45.21%)
		**Due to differences in characteristics (in %)**		**Due to differences in coefficients (in %)**	
Age		−0.16	−2.99 ***	−4.64	27.00
Males		0.13 **	0.11 **	−15.35 ***	−19.37 ***
Married		0.76	0.38	6.03	−4.90
High educational status		−4.87 ***	−9.26 ***	23.35 ***	53.51 ***
Medium educational status		−3.14 ***	−2.97 ***	32.52 ***	79.20 ***
Employed		0.18	−0.42 **	30.77 **	53.03 ***
Out of labour force		0.05	0.02	17.84	27.96 **
High income		0.05	0.04	1.05	−10.11
Middle income		0.00	0.10	−3.66	−8.56
Willingness to pay for healthcare		−0.40 ***	−1.24 ***	4.66	10.91 ***
Doctor trust		11.92 ***	13.14 ***	43.29 ***	−1.70
Self-assessed health status		3.13 ***	0.10 ***	−5.36	9.55
Health expenditure (% of GDP)			12.59 ***		36.53
Potential Years of Life Lost (per 100,000 persons)			45.90 ***		−91.65 ***
Observations		53,500	53,500	53,500	

Note: ** *p* < 0.05; *** *p* < 0.01. All models include country dummies.

**Table 8:** Determinants of individual perceptions about healthcare systems efficiency. Anglo-world sample. Oaxaca decomposition.

	Groups	2011–2013 vs. 2021–2023			
Variable Names					
Gap in individual perceptions about healthcare systems efficiency between the two waves		0.077 ***	0.077 ***	0.077 ***	0.077 ***
Characteristics (explained difference)		−0.004 (−5.18%)	−0.051 (−66.63%)	−0.004 (−5.18%)	−0.051 (−66.63%)
Coefficients (unexplained difference)		0.008 *** (105.18%)	0.129 *** (166.63%)	0.008 *** (105.18%)	0.129 *** (166.63%)
		**Due to differences in characteristics (in %)**		**Due to differences in coefficients (in %)**	
Age		−2.19	−2.40 **	109.63	41.11
Males		0.08	0.12	3.22	6.63
Married		−2.81	−1.03	−19.35	−33.62
High educational status		2.84	4.32	−36.97	−28.40
Medium educational status		−0.20	−3.46 **	−20.76	−8.03
Employed		2.01	4.34	83.20	82.04
Out of labour force		−0.55	−2.86	60.92	70.39
High income		−0.36	−0.22	−8.93	−5.02
Middle income		0.02	−0.09	−11.51	−13.01
Willingness to pay for healthcare		0.98 ***	0.94 ***	−9.66	−10.90
Doctor trust		−1.84 ***	−1.84 ***	3.46	−26.05
Self-assessed health status		−3.11 ***	−2.75 ***	9.46	15.74
Health expenditure (% of GDP)			−62.04 ***		−505.97 ***
Potential Years of Life Lost (per 100,000 persons)					
Observations		5018	5018	5018	5018

Note: ** *p* < 0.05; *** *p* < 0.01. All models include country dummies. Potential years of life lost are omitted from regressions due to collinearity.

**Table 9:** Determinants of individual perceptions about healthcare systems efficiency. European sample. Oaxaca decomposition.

	Groups	2011–2013 vs. 2021–2023			
Variable Names					
Gap in individual perceptions about healthcare systems efficiency between the two waves		0.043 ***	0.043 ***	0.043 ***	0.043 ***
Characteristics (explained difference)		0.007 *** (15.85%)	0.071 *** (162.86%)	0.007 *** (15.85%)	0.071 *** (162.86%)
Coefficients (unexplained difference)		0.037 *** (84.15%)	−0.027 *** (−62.86%)	0.037*** (84.15%)	−0.027 *** (−62.86%)
		**Due to differences in characteristics (in %)**		**Due to differences in coefficients (in %)**	
Age		0.80	−1.69	15.13	−11.63
Males		0.10	0.07	−34.51 ***	−24.21 ***
Married		1.33 ***	0.41	8.87	−0.14
High educational status		−4.79	−9.95 ***	92.70 ***	58.29 ***
Medium educational status		−8.54 ***	−4.12 ***	129.30 ***	74.09 ***
Employed		−0.68	−0.62	52.79	43.00
Out of labour force		−0.36	−0.19	26.65	19.80
High income		0.00	0.02	−3.75	−8.67
Middle income		0.24	0.20	−16.49 ***	−1.00
Willingness to pay for healthcare		−0.86	−0.55	1.45	0.99
Doctor trust		26.99 ***	16.41 ***	26.25	1.16
Self-assessed health status		1.50 ***	0.73 ***	−3.42	9.39
Health expenditure (% of GDP)			173.57 ***		−606.97 ***
Potential Years of Life Lost (per 100,000 persons)			−10.22		−212.94 ***
Observations		34,897	34,897	34,897	34,897

Note: *** *p* < 0.01. All models include country dummies.

**Table 10:** Determinants of individual perceptions about healthcare systems efficiency. East Asia sample. Oaxaca decomposition.

	Groups	2011–2013 vs. 2021–2023	
Variable Names			
Gap in individual perceptions about healthcare systems efficiency between the two waves		0.152 ***	
Characteristics (explained difference)		0.009 *** (5.75%)	
Coefficients (unexplained difference)		0.143 *** (94.25%)	
		**Due to differences in characteristics (in %)**	**Due to differences in coefficients (in %)**
Age		2.77 ***	−46.59 ***
Males		0.34 **	1.82
Married		−0.17	0.06
High educational status		−5.19 ***	−2.81
Medium educational status		0.11 ***	−2.11
Employed		1.11	−17.47
Out of labour force		−1.75	−1.67
High income		0.13	9.06 **
Middle income		0.03	7.46
Willingness to pay for healthcare		0.86 ***	−0.80
Doctor trust		6.19 ***	30.48 ***
Self-assessed health status		1.04	1.54
*Observations*		13,585	13,585

*Note*: ** *p* < 0.05; *** *p* < 0.01. All models include country dummies. Data on healthcare expenditure and potential years of life lost are not available for China and Taiwan.

## Data Availability

The data are publicly available from ISSP Research Group (2024): International Social Survey Programme: Health and Health Care I-II—Cumulation. GESIS, Cologne. Z8794 Data file Version 1.0.0, http://dx.doi.org/10.4232/1.14438.

## References

[1] Borzuchowska M., Kilańska D., Kozłowski R., Iltchev P., Czapla T., Marczewska S., Marczak M. (2023). The effectiveness of healthcare system resilience during the COVID-19 pandemic: A case study. Medicina.

[2] Halma M.T.J., Guetzkow J. (2023). Public health needs the public trust: A pandemic retrospective. BioMed.

[3] Biernacki M. (2025). Assessment of the efficiency and effectiveness of health care systems in European Union countries before and during the COVID-19 pandemic. Front. Public Health.

[4] Manavgat G., Audibert M. (2024). Healthcare system efficiency and drivers: Re-evaluation of OECD countries for COVID-19. SSM-Health Syst..

[5] Baumann L.A., Reinhold A.K., Levke Brütt A. (2022). Public and patient involvement in health policy decision-making on the health system level—A scoping review. Health Policy.

[6] Trein P., Fuino M., Wagner J. (2021). Public opinion on health care and public health. Prev. Med. Rep..

[7] Ellis L.A., Dammery G., Gillespie J., Ansell J., Wells L., Smith C.L., Wijekulasuriya S., Braithwaite J., Zurynski Y. (2024). Public perceptions of the Australian health system during COVID-19: Findings from a 2021 survey compared to four previous surveys. Health Expect..

[8] İzmir O., Lebcir R.M., Oypan O. (2025). Exploring pandemic preparedness through public perception and its impact on health service quality, attitudes, and healthcare image. Sci. Rep..

[9] Gilson L. (2003). Trust and the development of health care as a social institution. Soc. Sci. Med..

[10] Buzelli L., Cameron G., Gardner T. (2022). Public Perceptions of the NHS and Social Care: Performance, Policy and Expectations (Policy Briefing).

[11] García Balaguera C., García O.Y., Gutiérrez M.V. (2022). Public perception of healthcare system response to COVID-19: Findings from a web-based observational study in Villavicencio, Colombia. PLoS Glob. Public Health.

[12] Melnyk M., Blyznyukov A., Kolomiiets S., Dinits R. (2024). Socio-economic determinants of public healthcare. Health Econ. Manag. Rev..

[13] Ren M., Zhang H., Meltzer D., Arora V.M., Prochaska M. (2023). Changes in patient perceptions of the provider most involved in care during COVID-19 and corresponding effects on patient trust. J. Patient Exp..

[14] Robert S.A., Booske B.C. (2011). US opinions on health determinants and social policy as health policy. Am. J. Public Health.

[15] Antinyan A., Bassetti T., Corazzini L., Pavesi F. (2021). Trust in the health system and COVID-19 treatment. Front. Psychol..

[16] Vasilescu M.D., Apostu S.A., Militaru E., Hysa E. (2022). Public opinion on European health policy: Lessons from the COVID-19 pandemic. Int. J. Environ. Res. Public Health.

[17] Blinder A.S. (1973). Wage discrimination: Reduced form and structural estimates. J. Hum. Resour..

[18] Oaxaca R. (1973). Male-female wage differentials in urban labor markets. Int. Econ. Rev..

[19] Jann B. (2008). The Blinder–Oaxaca decomposition for linear regression models. Stata J..

[20] Powers D.A., Yoshioka H., Yun M.S. (2011). mvdcmp: Multivariate decomposition for nonlinear response models. Stata J..

[21] Sia D., Onadja Y., Nandi A., Foro A., Brewer T. (2014). What lies behind gender inequalities in HIV/AIDS in sub-Saharan African countries: Evidence from Kenya, Lesotho and Tanzania. Health Policy Plan..

[22] Tzogiou C., Boes S., Brunner B. (2021). What explains the inequalities in health care utilization between immigrants and non-migrants in Switzerland?. BMC Public Health.

[23] Abdulloev I., Gang I.N., Yun M.-S. (2014). Migration, education and the gender gap in labour force participation. Eur. J. Dev. Res..

[24] Rahimi E., Hashemi Nazari S.S. (2021). A detailed explanation and graphical representation of the Blinder–Oaxaca decomposition method with its application in health inequalities. Emerg. Themes Epidemiol..

[25] Gardeazabal J., Ugidos A. (2004). More on identification in detailed wage decompositions. Rev. Econ. Stat..

[26] Yun M.-S. (2004). Decomposing differences in the first moment. Econ. Lett..

[27] Yun M.-S. (2005). A simple solution to the identification problem in detailed wage decompositions. Econ. Inq..

[28] Goldstein J.R., Lee R.D. (2020). Demographic perspectives on the mortality of COVID-19 and other epidemics. Proc. Natl. Acad. Sci. USA.

[29] Pifarré i Arolas H., Acosta E., López-Casasnovas G., Lo A., Nicodemo C., Riffe T., Myrskylä M. (2021). Years of life lost to COVID-19 in 81 countries. Sci. Rep..

[30] Rahotă D.M., Țîrț D.P., Daina L.G., Daina C.M., Ilea C.D.N. (2024). Using Potential Years of Life Lost (PYLL) to Compare Premature Mortality between Romanian Counties to Confirmed COVID-19 Cases in 2020 and 2021. Healthcare.

[31] Woolf S.H., Chapman D.A., Sabo R.T., Weinberger D.M., Hill L., Taylor D.D.H. (2020). Excess deaths from COVID 19 and other causes. JAMA.

[32] Blendon R.J., Brodie M., Benson J.M., Altman D.E., Buhr T. (2006). Americans’ views of health care costs, access, and quality. Milbank Q..

[33] Calder R., Dunkin R., Rochford C., Nichols T. (2019). Australian health services: Too complex to navigate. A Review of the National Reviews of Australia’s Health Service Arrangements (Policy Issues Paper No. 1).

[34] Moucheraud C., Guo H., Macinko J. (2021). Trust in governments and health workers low globally, influencing attitudes toward health information, vaccines. Health Aff..

[35] Busemeyer M.R. (2021). Health Care Attitudes and Institutional Trust During the COVID-19 Crisis: Evidence from the Case of Germany (Working Paper No. 01).

[36] Borisova L.V., Martinussen P.E., Rydland H.T., Stornes P., Eikemo T.A. (2017). Public evaluation of health services across 21 European countries: The role of culture. Scand. J. Public Health.

[37] Cavazza N., Roccato M. (2025). Personal experiences with the national healthcare system and institutional trust in times of COVID-19. Political Psychol..

[38] Mattila M., Rapeli L. (2018). Just sick of it? Health and political trust in Western Europe. Eur. J. Political Res..

[39] Menon A., Kavanagh N.M., Falkenbach M., Wismar M., Greer S.L. (2025). The role of health and health systems in shaping political engagement and rebuilding trust in democratic institutions. Lancet Reg. Health-Eur..

[40] Mazza J., Scipioni M. (2022). The Brief Rally Around the Flag Effect of COVID-19 in Europe (JRC—Joint Research Centre Technical Report).

[41] Kritzinger S., Foucault M., Lachat R., Partheymüller J., Plescia C., Brouard S. (2021). ‘Rally round the flag’: The COVID-19 crisis and trust in the national government. West Eur. Politics.

[42] van der Meer T., Steenvoorden E., Ouattara E. (2023). Fear and the COVID-19 rally round the flag: A panel study on political trust. West Eur. Politics.

[43] Singh S.R. (2014). Public health spending and population health: A systematic review. Am. J. Prev. Med..

[44] Zhu Y., Li Y., Wu M., Fu H. (2022). How do Chinese people perceive their healthcare system? Trends and determinants of public satisfaction and perceived fairness, 2006–2019. BMC Health Serv. Res..

[45] World Health Organization (WHO) (2024). Report Measuring and Maximizing Public Support for Health Policies: Behavioural and Cultural Insights Policy Brief Series.

